# Characterization of the expression of *Salmonella *Type III secretion system factor PrgI, SipA, SipB, SopE2, SpaO, and SptP in cultures and in mice

**DOI:** 10.1186/1471-2180-9-73

**Published:** 2009-04-17

**Authors:** Hao Gong, Jing Su, Yong Bai, Lu Miao, Kihoon Kim, Yonghua Yang, Fenyong Liu, Sangwei Lu

**Affiliations:** 1 Division of Infectious Diseases, School of Public Health, 16 Barker Hall, University of California, Berkeley, CA 94720; 2 State Key Laboratory of Pharmaceutical Sciences, School of Life Sciences, Nanjing University, Nanjing, Jiangsu, 210093, PR China

## Abstract

**Background:**

The type III secretion systems (T3SSs) encoded by *Salmonella *pathogenicity island 1 and 2 (SPI-1 and SPI-2) are important for invasion of epithelial cells during development of *Salmonella*-associated enterocolitis and for replication in macrophages during systemic infection, respectively. *In vitro *studies have previously revealed hierarchical transport of different SPI-1 factors and ordered synergistic/antagonistic relationships between these proteins during *Salmonella *entry. These results suggest that the level and timing of the expression of these proteins dictate the consequences of bacterial infection and pathogenesis. However, the expression of these proteins has not been extensively studied *in vivo*, especially during the later stages of salmonellosis when the infection is established.

**Results:**

In this study, we have constructed bacterial strains that contain a FLAG epitope inserted in frame to SPI-1 genes *prgI*, *sipA*, *sipB*, *sopE2*, *spaO*, and *sptP*, and investigated the expression of the tagged proteins both *in vitro *and *in vivo *during murine salmonellosis. The tagged *Salmonella *strains were inoculated intraperitoneally or intragastrically into mice and recovered from various organs. Our results provide direct evidence that PrgI and SipB are expressed in *Salmonella *colonizing the spleen and cecum of the infected animals at early and late stages of infection. Furthermore, this study demonstrates that the SpaO protein is expressed preferably in *Salmonella *colonizing the cecum but not the spleen and that SptP is expressed preferably in *Salmonella *colonizing the spleen but not the cecum.

**Conclusion:**

These results suggest that *Salmonella *may express different SPI-1 proteins when they colonize specific tissues and that differential expression of these proteins may be important for tissue-specific aspects of bacterial pathogenesis such as gastroenterititis in the cecum and systemic infection in the spleen.

## Background

*Salmonella enterica *is among the most important and common etiological factors of food-borne disease [1-3]. Its infection causes a diverse range of diseases from mild self-limiting gastroenterititis to fatal systemic typhoid fever. *S. enterica *serovar Typhimurium, which can lead to various diseases in different hosts [4], is an important source of bacterial poisoning of contaminated food and water. Infection of humans with *S. typhimurium *usually causes self-limiting enterocolitis, but there are serious consequences when systemic invasion occurs. Systemic infection in sensitive mice somewhat simulates the pathological process of typhoid fever in human patients and it is thus an appropriate model to assess gene expression associated with invasiveness as well as colonization [4]. Understanding the process of bacterial infection and pathogenesis is central in developing novel strategies and new compounds for the treatment of diseases associated with *Salmonella *infection.

Two hallmarks of *Salmonella *pathogenesis are the invasion of non-phagocytic cells such as epithelial cells of the intestinal mucosa in self-limiting enterocolitis, and the survival and replication inside infected macrophages during systemic infection. The mechanisms of both processes are linked to the functions of two type III secretion systems (T3SS) for virulence proteins of *Salmonella *[5]. T3SSs are specialized protein secretion systems encoded by gene clusters in the genomes of many pathogenic bacteria [6-8]. One of these T3SSs is encoded by a cluster of virulence genes termed *Salmonella *Pathogenicity Island 1 (SPI-1). The second T3SS is encoded by another cluster of genes in a separate pathogenicity island termed *Salmonella *Pathogenicity Island 2 (SPI-2). Each of the T3SSs is constituted by a secretome (secretion apparatus), its substrates (effector proteins) and chaperone proteins [7,9]. These two T3SSs perform quite different functions in *Salmonella *infection. It is generally believed that SPI-1 T3SS is responsible for invasion of non-phagocytic cells, while SPI-2 T3SS is essential for the intracellular replication and systemic infection [7,9]. In addition to the well-characterized SPI-1 and SPI-2, many other SPIs have been described in *Salmonella *but their roles have not yet been fully investigated [10-12]. Chracterization of the expression patterns of the genes of SPI-1 and other SPIs should provide insight into the functional roles of these factors in *Salmonella *infection.

The modulation of expression of genes in SPI-1 is remarkably complex and needs further characterization [13,14]. For example, in contrast to the current model of SPI-mediated pathogenesis, several studies have shown that the expression of some SPI-1 genes is induced upon invasion of both macrophages and epithelial cells and that several SPI-1 factors are essential for intracellular replication [15-17]. Furthermore, SPI-1 proteins, SipA, SopA, SopB, SopD, and SopE2 were found to be expressed by *Salmonella *in infected animals at the late stages of infection [17]. These results suggest that in addition to its generally recognized role in invasion, the SPI-1 factors may play an important role post-invasion. Hence, the role of the SPI-1 factors in bacterial pathogenesis, especially during the late stages of salmonellosis, needs further characterization and their expression *in vivo *needs to be studied.

Extensive studies have been carried out to investigate the expression of SPI-1 under different conditions *in vitro *[13,18]. However, most of these studies were performed by examining the transcription levels of these genes either using microarray or a reporter system [18-20], and protein expression under the native promoter for these T3SS factors has not been extensively investigated. In addition, little is known about the expression of these factors *in vivo*, especially during the established phase of infection.

In this study, we constructed *Salmonella *strains that contained a FLAG epitope sequence inserted in frame into the carboxyl terminus of SPI-1 genes *prgI*, *sipA*, *sipB*, *sopE2*, *spaO*, and *sptP*, and characterized the expression of the tagged proteins *in vitro *and *in vivo *during murine salmonellosis. The FLAG epitope is an octapeptide protein tag that has been widely used for tagging a protein, which in turn can be detected and studied using the anti-FLAG antibody [21]. The mutants were inoculated intraperitoneally or intragastrically into mice and recovered from various organs. Our results provide direct evidence that PrgI and SipB are expressed *in vivo *at both the early and late stages of bacterial infection. Furthermore, this study demonstrates that the SpaO protein is preferably expressed in *Salmonella *colonizing the cecum and that SptP is preferably expressed in *Salmonella *colonizing the spleen. These results further suggest that different SPI-1 proteins are expressed by *Salmonella *when they colonize specific tissues and that differential expression of these proteins may play an important role in bacterial pathogenesis in specific tissues.

## Results

### Wild type-like growth phenotypes of the tagged strains *in vitro *and *in vivo*

**Figure 1:**
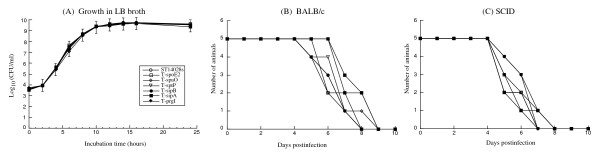
**Growth curve analysis of different bacterial strains in LB broth (A) and mortality of the BALB/c (B) and SCID mice (C) infected with the ST14028s strain, T-prgI, T-spoE2, T-spaO, T-sptP, T-sipB, and T-sipA**. BALB/c mice (B) and CB17 SCID mice (C) (5 animals per group) were infected intragastrically with 5 × 10^6 ^and 1 × 10^3 ^CFU of each bacterial strain, respectively.

Bacterial strains T-prgI, T-sipA, T-sipB, T-sopE2, T-spaO, and T-sptP were derived from the wild type *Salmonella *strain (ST14028s) by inserting the FLAG epitope tag sequences into SPI-1 ORFs *prgI, sipA*, *sip*B, *sopE*2, *spaO*, and *sptP*, respectively (Table 1). One of our main objectives in the study was to use the expression of the tagged proteins as a model to monitor the corresponding proteins during *Salmonella *infection. Thus, it is necessary to determine whether the tagged strains retain the growth and virulence properties of the parental (wild type) ST14028s strain both *in vitro *and *in vivo*. In our *in vitro *growth study, growth curve analyses showed that all the tagged strains grew as well as ST14028s in LB broth (Figure 1A), suggesting that the insertion of the tag sequence did not significantly affect bacterial growth *in vitro *[17].

**Table 1:** The bacterial strains and plasmid constructs used in the study

Bacterial strains, plasmids		Description	Reference/source
*S. typhymurium *strains	ST14028s	Wild type and parental strain	
	
	T-prgJ	prgJ::1xFLAG	This study
	
	T-sipA	sipA::1xFLAG	This study
	
	T-sipB	sipB::1xFLAG	This study
	
	T-sopE2	sopE2::1xFLAG	This study
	
	T-spaO	spaO::1xFLAG	This study
	
	T-sptP	sptP::1xFLAG	This study

*E. coli s*train	DH5a	F^-^Φ80d*lac*ZΔM15Δ(*lac*ZYA-*argF*)*U169deoRrecA1endA1hsdR17*(r_k_-m_k_^+^)*phoAsupE44λ*^-^*thi-1gyrA96relA1*	Invitrogen

Plasmids	pUC-H1PF1	Ap^r ^and Kan^r^, template plasmid for 1xFLAG epitope tag	[43]
	
	Kan-clone7 plasmid	Derived from pkD4, containing a kanamycin resistance cassette and sequence which can be recognized by flapase	[44]
	
	pkD46	Ap^r^, containing the Red recombinase of λ phage	[44]
	
	pCP20	Containing the expression cassette of flapase which can remove the kanamycin resistance cassette from the mutant strains	[44]

Both immunocompetent BALB/c mice and immunodeficient CB17 SCID mice were used in our study to investigate the pathogenesis and virulence of the constructed *Salmonella *strains. Immunodeficient animals, such as the CB17 SCID mice that lack functional T and B lymphocytes, are extremely susceptible to *Salmonella *infection. Analysis of bacterial growth in these mice serves as an excellent model for comparing the virulence of different bacterial strains and mutants and for studying how they cause opportunistic infections in immunocompromised hosts. Mice were inoculated intraperitoneally to assess the ability of the bacteria to cause systemic infection. Mice were also infected intragastrically, a natural route of infection. To study the virulence of the tagged strains, the survival rates of the infected animals were determined. When intragastrically infected with 5 × 10^6 ^CFU of the tagged or the wild type strains, all BALB/c mice died within 9 days postinfection (Figure 1B). When SCID mice were infected intragastrically with 1 × 10^3 ^CFU bacteria, all animals died within 8 days postinfection (Figure 1C). In either strain of mice, no difference was observed between the wild type and tagged strains (Figure 1B–C), suggesting that epitope tagging of the SPI-1 proteins did not affect the virulence of the *Salmonella *strains. Similar results were also observed when animals were intraperitoneally infected with the strains (data not shown).

To study the pathogenesis of the tagged strains, the colonization of spleen, liver, and cecum was determined at different time points after infection. No significant differences in the colonization of the internal organs were observed between the parental (wild type) ST14028s strain and the tagged bacterial strains, regardless of the route of inoculation (Table 2). These results suggest that tagging of the target ORF does not impair the invasiveness, growth, and virulence of the bacteria, and that the tagged strains can be used as model strains to study infection of *Salmonella in vitro *and *in vivo*, including the expression of SPI-1 factors.

**Table 2:** The numbers of bacteria (CFU) in different organs from animals

*Salmonella *strains		Colonization (i.p.)		Colonization (i.g.)	
		
		log CFU per organ		log CFU per organ	
		
		Liver	Spleen	Spleen	Cecum
(A) BALB/c mice	ST14028s	8.5 ± 0.5	7.7 ± 0.5	7.3 ± 0.5	7.5 ± 0.3
	
	T-prgJ	8.6 ± 0.5	7.9 ± 0.6	7.1 ± 0.5	7.5 ± 0.7
	
	T-sipA	9.4 ± 0.5	8.4 ± 0.7	7.4 ± 0.5	7.5 ± 0.7
	
	T-sipB	8.4 ± 0.5	7.5 ± 0.5	7.3 ± 0.5	7.7 ± 0.3
	
	T-sopE2	8.8 ± 0.5	8.3 ± 0.7	7.5 ± 0.5	7.4 ± 0.7
	
	T-spaO	8.5 ± 0.5	7.6 ± 0.8	7.0 ± 0.5	6.9 ± 0.6
	
	T-sptP	8.5 ± 0.5	7.6 ± 0.5	7.0 ± 0.5	6.9 ± 0.6

(B) SCID mice	ST14028s	8.7 ± 0.5	7.7 ± 0.5	7.9 ± 0.5	8.2 ± 0.6
	
	T-prgJ	8.9 ± 0.5	7.6 ± 0.7	7.3 ± 0.7	8.4 ± 0.6
	
	T-sipA	8.6 ± 0.5	7.5 ± 0.6	7.8 ± 0.6	8.9 ± 0.7
	
	T-sipB	8.9 ± 0.5	8.3 ± 0.5	7.6 ± 0.5	8.8 ± 0.7
	
	T-sopE2	8.9 ± 0.5	8.3 ± 0.6	7.6 ± 0.7	8.8 ± 0.4
	
	T-spaO	8.5 ± 0.5	8.0 ± 0.7	8.5 ± 0.7	8.6 ± 0.5
	
	T-sptP	8.9 ± 0.5	8.3 ± 0.6	7.6 ± 0.5	8.8 ± 0.5

*Mice were either infected intraperitoneally (i.p.) or intragastrically (i.g.) with 1 × 10^5 ^CFU for BALB/c mice or 1 × 10^2 ^CFU for SCID mice. A group of 5 mice was infected and the organs were harvested at 5 (for i.p. infection) or 7 days (for i.g. inoculation) post infection. Each sample was analyzed in triplicate and the analysis was repeated at least twice. The CFU of the sample was expressed as the average of the values obtained. The concentrations of bacteria were recorded as CFU/ml of organ homogenate. The limit of bacteria detection in the organ homogenates was 10 CFU/ml.

### *In vitro *synthesis and secretion of the tagged SPI-1 proteins under different cultured conditions

After being ingested from contaminated food or polluted water, *Salmonella *will encounter a series of extreme environmental changes such as acidity in the stomach, hypoxia, hyperosmolarity, and other conditions such as fermentation in the gut [22-24]. The expression of bacterial genes including those of SPI-1 is expected to be regulated to allow bacteria to adapt to new environments and to prepare for the invasion of the intestinal epithelium. To investigate the synthesis and secretion of the SPI-1 proteins, each of the tagged strains was grown under five different conditions that resembled the early stages of its natural infection, and the expression of the tagged proteins was studied.

#### (A) Expression in rich media LB broth

**Figure 2:**
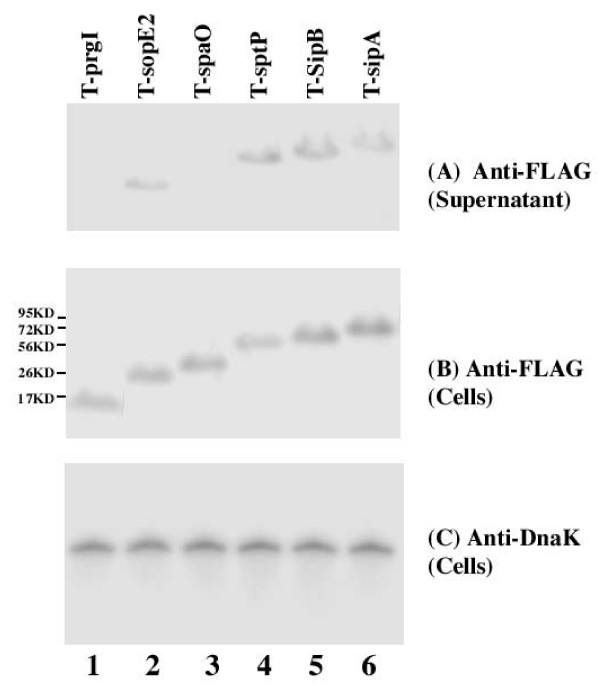
**Western analyses of the synthesis (B) and secretion (A) of the tagged proteins from bacterial strains T-prgI (13 KD)(lane 1), T-spoE2 (29 KD) (lane 2), T-spaO (36 KD) (lane 3), T-sptP (62 KD) (lane 4), T-sipB (65 KD) (lane 5), and T-sipA (76 KD) (lane 6)**. The expression of bacterial DnaK was used as the internal control (C). Protein samples were separated in SDS-polyacrylamide gels and reacted with antibodies against the FLAG sequence (A-B) and DnaK (C). Each lane was loaded with material from 5 × 10^7 ^CFU bacteria. The molecular masses of some of the proteins in the PageRuler protein size markers (Fermentas) are shown and given in kiloDaltons (KD).

Western analyses were carried out to detect the expression of the tagged proteins with an anti-FLAG antibody (Figure 2A and 2B), using the expression of bacterial DnaK protein as the internal control (Figure 2C). Normalization of samples was also carried out by loading total protein extracted from the same CFU (e.g. 5 × 10^7 ^CFU) of bacteria in each lane. SPI-1 proteins PrgI, SopE2, SpaO, SptP, SipB, and SipA were detected in *Salmonella *cultured in LB broth (Figure 2B). Furthermore, SpoE2, SptP, SipB, and SipA but not PrgI or SpaO were detected in the culture supernatant (Figure 2A, data not shown). These results are consistent with the previous observations that SpaO and PrgI are the structural components of the needle complex [5].

#### (B) Expression under different pH conditions

**Figure 3:**
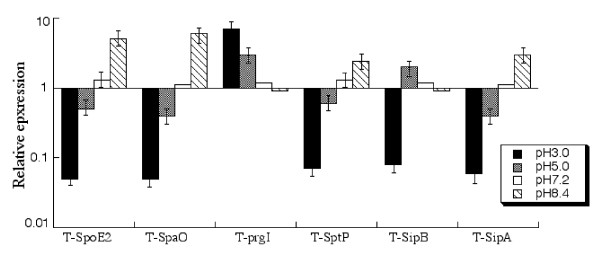
**Effect of pH values on the expression of the tagged SPI-1 proteins**. Cultures of the tagged strains T-spoE2, T-spaO, T-prgI, T-sptP, T-sipB, and T-sipA were grown in the presence of culture media at pH3.0, 5.0, 7.0, 7.2, and 8.4, as described in Methods and Materials. The values of the relative expression, which are the means from triplicate experiments, represent the ratios for the level of the tagged protein under the pH conditions to the control pH7.0 condition. The standard deviation is indicated by the error bars.

Acidity in the stomach is the first stress *Salmonella *meets after being ingested orally. Because the environment in the intestine is relatively basic, *Salmonella *will encounter an increase in pH after it reaches the intestine. It is believed that few SPI-1 proteins are expressed in the stomach and increasing pH levels from acidic conditions in the stomach to the conditions in the intestine could induce the expression of SPI-1 proteins [5,13]. To determine the effect of pH values on the expression of the tagged ORFs, bacterial strains were grown under different pH conditions. Figure 3 summarizes the results of the effect of pH on the expression of SPI-1 proteins. These results indicated that the expression of the tagged SPI-1 proteins, except PrgI and SipB, was down-regulated at low pH (e.g. pH3.0 and pH5.0) and that neutral and basic conditions (i.e. pH7.2 and pH8.4) induced the expression of SPI-1 proteins. In contrast, SipB had the highest expression at pH5.0. PrgI had the highest expression at pH 3.0 compared to that at pH5.0 and pH7.0 (Figure 3), suggesting that this protein may be expressed at a considerable level as early as in the stomach during *Salmonella *infection *in vivo*.

#### (C) Effect of osmolarity on the expression

High osmolarity is one of the environmental stresses that bacteria encounter in the intestines. Previous reports indicated that osmolarity was an independent factor affecting the virulence of several bacterial pathogens in the gut and that high osmolarity may promote *Salmonella *adhesion and invasion to intestinal epithelial cells [22]. Recently, it has been reported that the transcription levels of SPI-1 genes *sipB*, *sipC*, and *sipD *are significantly enhanced in the presence of high osmolarity (e.g. 300 mM NaCl) in a genome-wide scanning experiment using *Salmonella *nucleotide microarray [19,24]. However, the effect of the osmolarity on the protein expression of SPI-1 factors has not been extensively investigated [25].

**Figure 4:**
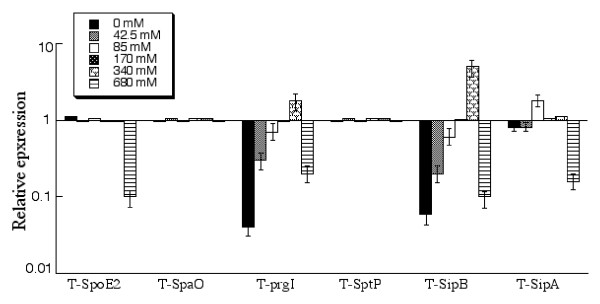
**Effect of osmolarity on the expression of the tagged SPI-1 proteins**. Cultures of the tagged strains T-spoE2, T-spaO, T-prgI, T-sptP, T-sipB, and T-sipA were grown in the presence of culture media under different concentrations of NaCl, as described in Methods and Materials. The values of the relative expression, which are the means from triplicate experiments, represent the ratios of the level of each protein under different osmolarity conditions to the control condition of 170 mM NaCl of the regular LB broth.

To test the influence of osmolarity on the protein levels of SPI-1 factors, bacterial strains were grown in the presence of different concentrations of NaCl. The expression of the tagged proteins was determined using Western analyses and the results are summarized in Figure 4. Osmolarity appeared to have no significant impact on the expression of SpaO and SptP. Higher osmolarity of up to 340 mM NaCl favored the expression of PrgI and SipB, while the very high concentration of NaCl at 680 mM inhibited the expression of SopE2 (Figure 4).

#### (D) Effect of oxygen limitation

**Figure 5:**
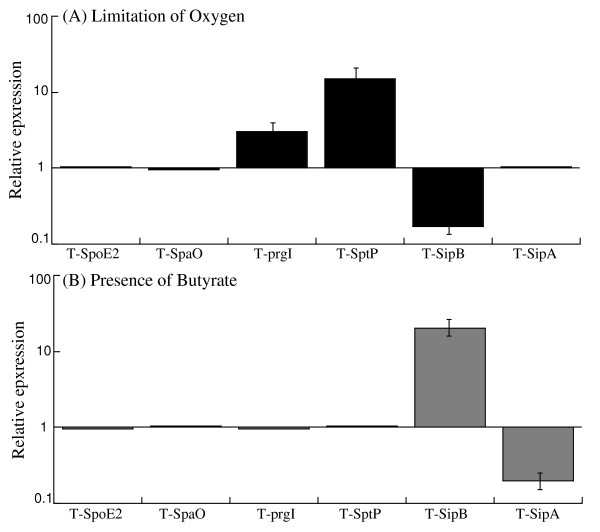
**Effect of the limitation of oxygen (A) and the presence of butyrate (B) on the expression of the tagged SPI-1 proteins**. Cultures of the tagged strains T-spoE2, T-spaO, T-prgI, T-sptP, T-sipB, and T-sipA were grown in the presence and limitation of oxygen (A), or the absence and presence of 10 mM butyrate (B), as described in Methods and Materials. The values of the relative expression, which are the means from triplicate experiments, represent the ratios for the levels of the tagged protein under the limitation of oxygen conditions to the control condition (i.e. in the presence of oxygen) (A) or the ratios for the levels of the proteins from the bacteria grown in the presence of 10 mM butyrate to those in the absence of butyrate (B).

A limited level of oxygen is an important characteristic of the environment in the intestine. It has been shown that oxygen limitation induces *Salmonella *invasiveness of intestinal mucosa while aerobic conditions render *Salmonella *less invasive [26,27]. Bajaj et al reported that the expression of the transcripts of six different SPI-1 invasion genes was coordinately regulated by oxygen, osmolarity, pH, PhoP/Q, and HilA [28]. In our experiments, oxygen limitation had little impact on the protein expression of SpoE2, SpaO, and SipA. In contrast, decreased levels of oxygen appeared to induce the protein expression of PrgI and SptP, but inhibited the expression of SipB (Figure 5A).

#### (E) Effect of butyrate

The anaerobic environment in the intestine favors bacterial fermentation. After bacteria reach the intestine, the fermentation process is initiated and three types of organic acids, acetate, propionate and butyrate accumulate [29]. These organic acids are important for maintaining the healthy status of the intestinal epithelium [29]. Limitation of butyrate could lead to intestinal inflammation and administration of butyrate could alleviate the severity of *Salmonella *infection of intestinal epithelium [30,31]. Recently, it has been reported that the transcription levels of 17 SPI-1 genes are down-regulated at least two-fold after *Salmonella *were exposed to 10 mM butyrate for 4 hours [20]. However, the effects of butyrate on protein levels of these factors have not been extensively studied. In our experiments, incubation with 10 mM butyrate does not significantly affect the protein levels of PrgI, SopE2, SpaO, and SptP (Figure 5B). In contrast, in the presence of butyrate, the protein level of SipB increased while that of SipA decreased.

### *In vivo *expression of the tagged SPI-1 proteins after intraperitoneal infection of *Salmonella*

To study the expression of SPI-1 proteins *in vivo *during systemic bacterial infection, BALB/c mice were infected intraperitoneally with different tagged strains. A high inoculum was used to ensure that sufficient bacteria would be recovered from internal organs. At different time points postinfection, mice were sacrificed and the spleen, stomach, and cecum were harvested. The numbers of bacteria in these three organs were determined. No bacteria were found in the stomach at 12–24 hours postinfection, consistent with the fact that the systemic *Salmonella *infection does not spread to the organ or is cleared at this early time point (data not shown).

**Figure 6:**
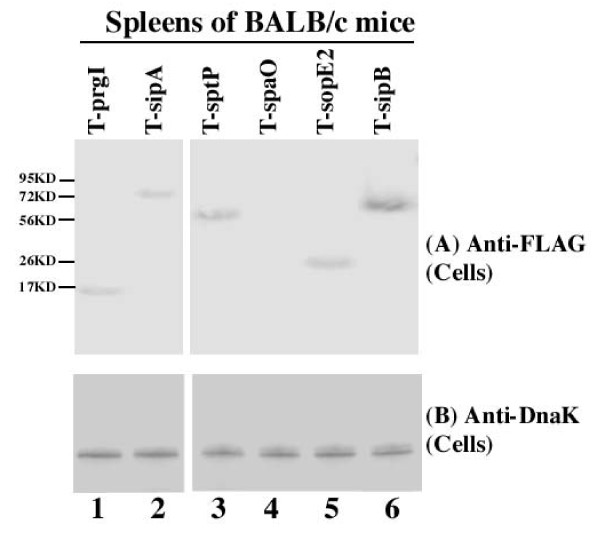
**Western analyses of the expression of the tagged proteins from the internalized bacterial strains T-prgI (lane 1), T-sipA (lane 2), T-sptP (lane 3), T-spaO (lane 4), T-sopE2 (lane 5), and T-sipB (lane 6) recovered from spleens**. BALB/c mice were intraperitoneally infected with 1 × 10^5 ^CFU of the tagged strains, and internalized bacteria were recovered from the spleens at 5 days post inoculation. The expression of bacterial DnaK was used as the internal control (B). Protein samples were reacted with antibodies against the FLAG sequence (A) and DnaK (B). Each lane was loaded with material from 5 × 10^7 ^CFU bacteria.

The expression of the tagged proteins in the bacterial strains isolated from the spleen and cecum of infected mice was detected using Western analysis with an anti-FLAG antibody and normalized using the expression of bacterial protein DnaK as the internal control (Figure 6A–B). Normalization of samples was also carried out by loading total protein extracted from the same CFU (e.g. 5 × 10^7 ^CFU) of bacteria in each lane. The protein level of DnaK did not appear to be significantly different in bacteria from the spleen and cecum as similar amount of the DnaK protein was detected from 5 × 10^7 ^CFU of each bacterial strain regardless of infection route (intraperitoneally or intragastrically) or time point postinfection (12–24 hours or 5–7 days) (data not shown).

**Figure 7:**
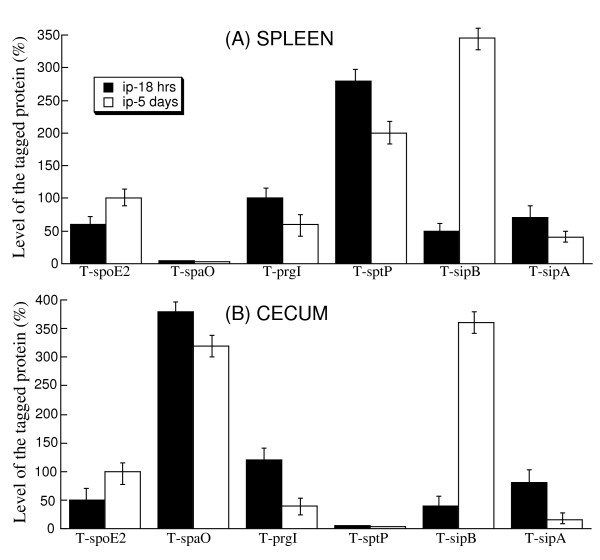
**Level of the tagged proteins from the internalized bacterial strains T-prgI, T-sipA, T-sptP, T-spaO, T-sopE2, and T-sipB recovered from the spleen (A) and cecum (B)**. BALB/c mice were intraperitoneally infected with 1 × 10^7 ^or 1 × 10^5 ^CFU of the tagged strains, and internalized bacteria were recovered from the spleen at 18 hours or 5 days post inoculation, respectively. The values, which are the means from triplicate experiments, represent the relative percentage of the level of the tagged proteins in the internalized bacteria recovered from the spleen (A) and cecum (B), as compared to the level of the tagged SpoE2 protein in the T-spoE2 recovered from the spleen and cecum at 5 days post inoculation, respectively.

*Salmonella *strains isolated from both the spleen and cecum at 18 hours postinfection continued to express PrgI, SpoE2, SipB, and SipA. In contrast, a substantial level of SpaO was detected in *Salmonella *isolated from the cecum but not the spleen, while that of SptP was observed in *Salmonella *recovered from the spleen but not the cecum (Figure 7A–B). These results suggest that SpaO and SptP are differentially expressed by *Salmonella *when they colonize specific organs and tissues.

To rule out the residual expression of the proteins from *in vitro *bacterial growth, we investigated infected mice for longer periods after infection. BALB/c mice were intraperitoneally infected with 1 × 10^5 ^CFU bacteria and tissues were collected at 5 days postinfection, prior to the onset of severe diseases associated with infection. Similar results were observed as described above for 18 hours postinfection, which showed that proteins PrgI, SopE2, SipB, and SipA were expressed in *Salmonella *isolated from both the spleen and cecum, and that SpaO and SptP were preferentially expressed by *Salmonella *recovered from the cecum and spleen, respectively (Figure 7). Indeed, the expression of the SpaO protein was more than 40 fold higher than that of SptP in *Salmonella *isolated from the cecum, while the SptP protein was expressed at least 70 times more than SpaO in *Salmonella *isolated from the spleen (Figure 7).

The protein levels of SopE2 and SipB at 5 days postinfection were higher than those at 18 hours postinfection in *Salmonella *isolated from both the cecum and spleen. In contrast, the levels of PrgI and SipA at 5 days postinfection were lower than those at 18 hours postinfection. It is interesting to note that the expression of SpaO in *Salmonella *colonizing the cecum and SptP in *Salmonella *colonizing the spleen decreased with the duration of the infection (Figure 7).

### *In vivo *expression of the tagged SPI-1 proteins during oral infection

**Figure 8:**
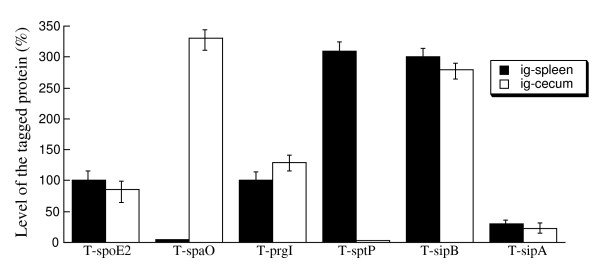
**Level of the tagged proteins from the internalized bacterial strains T-prgI, T-sipA, T-sptP, T-spaO, T-sopE2, and T-sipB recovered from the spleen and cecum**. BALB/c mice were intragastrically infected with 1 × 10^5 ^CFU of the tagged strains, and internalized bacteria were recovered from the spleen and cecum at 7 days post inoculation. The values, which are the means from triplicate experiments, represent the relative percentage of the level of the tagged proteins in the internalized bacteria recovered from the spleen and cecum, as compared to the level of the tagged SpoE2 protein in the T-spoE2 recovered from the spleens at 7 days post inoculation.

To study whether the tagged SPI-1 proteins are expressed during *Salmonella *infection acquired by the natural route, BALB/c mice were infected intragastrically with 1 × 10^5 ^CFU bacteria. Spleens and cecums were collected and the bacteria were recovered at day 7 postinfection, prior to the onset of severe diseases associated with infection. Similar to what was observed in *Salmonella *from intraperitoneally infected mice, PrgI, SopE2, SipB, and SipA were detected in *Salmonella *isolated from both the spleen and cecum, while SpaO and SptP were found to be expressed preferentially in *Salmonella *isolated from the cecum and spleen, respectively (Figure 8). Protein level of DnaK did not appear to be significantly different in bacteria recovered from the spleen and cecum (data not shown). These results provide direct evidence that PrgI and SipB are expressed *in vivo *in intragastrically-infected mice. Furthermore, these results suggest that the SpaO and SptP proteins are expressed preferably in *Salmonella *colonizing the cecum and spleen respectively during oral infection of mice.

## Discussion

Our results provide direct evidence that PrgI and SipB are expressed *in vivo *in both the early and late stages of *Salmonella *infection. Our data on the tagged SopE2 and SipA proteins are consistent with previous results that these proteins are expressed in infected animals during the late stages of salmonellosis [17]. Furthermore, this study demonstrates that SpaO and SptP are differentially expressed in *Salmonella *colonizing the cecum and spleen, respectively. These results further suggest that different SPI-1 proteins are expressed by *Salmonella *in specific tissues and that differential expression of these proteins may be important for bacterial pathogenesis in certain tissues such as gastroenterititis in the cecum and typhoid fever during systemic infection in the spleen.

It is possible that the observed expression of the tagged ORFs is due to adventitious mutations introduced during the construction and growth of the mutants *in vitro *and in animals, which may affect their expression. It is also conceivable that the function and expression of the ORFs can be affected by insertion of an epitope tag. Such an insertion may influence the function of other genes adjacent to the insertion region and therefore, possibly affect the expression of the tagged ORF. However, several lines of evidence strongly suggest that this is unlikely. All the tagged mutants grew as well as the wild type ST14028s strain *in vitro *in LB broth and *in vivo *in both BALB/c and SCID mice that were either infected intraperitoneally or intragastrically (Figure 1 and Table 2). Furthermore, the mutants exhibited similar virulence as the ST14028s strain. These results suggest that the FLAG epitope insertion does not affect the function of the tagged ORF, and that the insertion does not cause any adventitious mutations that may impact bacterial virulence and pathogenicity. Thus, the observed expressions of the tagged proteins from the bacterial strains are believed to represent the expression of the wild type SPI-1 proteins *in vitro *and *in vivo*.

Previous studies have shown that the SopE2 and SipA proteins are expressed in *Salmonella *isolated from the spleen [17]. Our results are consistent with these previous observations, and further demonstrate that these proteins are expressed in *Salmonella *isolated from the cecum. Our results also provide direct evidence that the PrgI and SipB proteins are expressed *in vivo*. PrgI is the component of the needle complex or "injectisome" that is traversed by a channel that serves as a conduit for the passage of proteins that travel the type III secretion pathway [5,32]. The needle, which is composed of a single protein, PrgI, is connected to a different substructure, the inner rod, which traverses the entire length of the base and is made of PrgJ. Recent evidences have suggested that the stoichiometry of PrgI and PrgJ, which is dictated by their protein expression levels, affects the length of the needle complex formed, and consequently, the ability of the bacteria to enter epithelial cells and induce cytotoxicity in macrophages [5,32,33]. Thus, the expression of PrgI protein is highly regulated and is essential for assembly of the secretion machinery. Interestingly, our results showed that PrgI was expressed efficiently at pH3.0 and the expression level was even higher than at pH5.0 and pH7.0 while all other SPI-1 proteins we studied were poorly expressed at pH3.0, suggesting that PrgI may be expressed early during oral infection and is available long before the assembly of the needle complex and the expression of other SPI-1 proteins.

The effector protein SipB is a *Salmonella *invasion protein (Sip) that is central to the initiation of the bacterial entry process. SipB and SipC form an extracellular complex following their secretion through the SPI-1 T3SS, and they are thought to assemble into a plasma membrane-integral structure (translocon) that mediates effector delivery [34-36]. Once delivered to the host cell membrane, they form a pore structure to facilitate effector transport [36]. In addition to its role as a component of the translocon, SipB has been reported to induce apoptosis of macrophages by associating with the proapoptotic protease caspase-1 [37]. These results suggest that the SipB protein has multiple functions that require highly regulated expression, including specific expression during the late stages of infection.

Our results demonstrate that SptP and SpaO are differentially expressed *in vivo *by *Salmonella *when they colonize the spleen and cecum, respectively. SptP encodes a multifunctional protein that primarily functions to reverse cellular changes (e.g. actin de-polymerization) stimulated by other effectors (e.g. SopE2) [5,38]. Its amino terminal domain encodes a GTPase activating protein (GAP) activity that antagonizes Rho-family GTPases including Rac1 and cdc42, while its carboxyl terminal region exhibits tyrosine phosphatase activity [5,38]. While the expression of SptP has been extensively studied *in vitro*, its expression *in vivo *has not been reported. The preferential expression of SptP by *Salmonella *colonizing the spleen but not the cecum suggests that the level of this protein is highly regulated *in vivo *and that appropriate level of expression may contribute to different consequences of pathogenesis. This is consistent with the recent observations that the GAP activity of SptP by itself was originally interpreted as an activity aimed at disrupting the actin cytoskeleton of the target cell; however, in the context of its delivery along with activators of Rho-family GTPases, the function of SptP proved to be the preservation of the actin cytoskeleton rather than its disruption [38-40]. Thus, examination of the expression of SptP and other secreted proteins *in vivo *in the context of infection, as reported in our study, is crucial and necessary to ultimately understand the actual function and action of these effectors.

Previous studies have shown that *Salmonella *mutants lacking *spaO *failed to assemble the needle complex, leading to its inability to secrete proteins and invade cells [41,42]. This suggests that the SpaO protein is essential for needle complex assembly and protein secretion critical for bacterial entry. However, its expression *in vivo *has not been reported. Our findings of the differential expression of SpaO preferentially by *Salmonella *colonizing the cecum but not spleen raises the possibility that efficient expression of this protein may not be needed by *Salmonella *in the spleen, possibly because bacteria entry can be accomplished with phagocytosis by splenic macrophages. Furthermore, tissue-specific differential regulation of the expression of SpaO, a protein essential for the secretion machinery [41,42], should provide another mechanism *in vivo *for the regulation of the amounts of effector proteins to be secreted into the host cells. Recent studies have revealed hierarchical transport of different effectors during *Salmonella *entry and extensive ordered synergistic and antagonist relationships between these effectors following their delivery into the host cells [5,39,40]. Thus, it is conceivable that differential expression of SpaO may dictate the amounts of needle complexes available during bacterial entry. This may result in hierarchical transport of specific effectors and specific functional interplay (synergistic or antagonist relationships) among these proteins in the host cells, leading to specific pathological consequences in different tissues.

We note that some of the protein expression results in our study may not be consistent with those from the expression of the transcripts of the SPI-1 genes that have been recently published [19,20]. The expression of SPI-1 genes is tightly controlled transcriptionally and post-transcriptionally [13]. Thus, we believe that our results of the SPI-1 protein expression *in vivo *may not necessarily correlate with the previous observations *in vitro*. Furthermore, the amounts of proteins expressed from the SPI-1 genes *in vivo *are in a delicate balance as there are hierarchical transports of different effectors during *Salmonella *entry and extensive ordered synergistic and antagonist relationships between these effectors following their delivery into the host cells. An imbalance of the amounts of these factors available during infection would seriously compromise the ability of the bacteria to establish successful infection. Our results complement and further extend previous findings of the expression of these SPI-1 factors, and demonstrate the importance of examining protein expression *in vivo *in the context of infection. Further investigation of the expression of these proteins as well as other bacterial proteins in different organs *in vivo *should provide significant insights into the role of these proteins in bacterial pathogenesis and their interactions with other bacterial and host proteins during *Salmonella *infection.

## Conclusion

By constructing *Salmonella *strains with a FLAG epitope sequence inserted in frame into the SPI-1 genes *prgI*, *sipA*, *sipB*, *sopE2*, *spaO*, and *sptP*, and characterizing the expression of the tagged proteins *in vitro *and *in vivo*, we provide direct evidence that PrgI and SipB are expressed *in vivo *in both the early and late stages of bacterial infection. Furthermore, this study demonstrates that the SpaO protein is preferentially expressed by *Salmonella *colonizing the cecum but not the spleen, and that SptP is preferentially expressed by *Salmonella *colonizing the spleen but not in the cecum. These results further suggest that different SPI-1 proteins are expressed by *Salmonella *when they colonize specific tissues and that differential expression of these proteins may play an important role in bacterial pathogenesis in specific tissues.

## Methods

### Bacterial strains and growth conditions

Bacterial strains and their genotypes are listed in Table 1. Strains were grown on LB agar or in LB broth. When necessary, the following antibiotics were added at the indicated concentrations: kanamycin, 50 μg/ml; ampicillin, 100 μg/ml.

Growth analysis of bacteria in LB broth was carried out by first inoculating one isolated colony in 2 ml LB broth and culturing at 37°C and 250 RPM overnight (about 16 hours). Thirty microliters of the overnight culture were then inoculated into 3 ml of LB broth and cultured at 37°C and 250 RPM. At time points of 0, 2, 4, 6, 8, 10, 12, 14, 16, and 24 hours after inoculation, 100 microliters of bacterial culture were collected and used for analysis by limiting dilution in sterile 96-well plates, and then plated on LB agar plates to determine their CFU (colony forming unit)/ml. Each sample was analyzed in triplicate and the analysis was repeated at least twice. The average value of CFU/ml was used to generate the growth curve.

### Construction of plasmids and tagged mutants

Plasmid constructs that were used in the study are listed in Table 1. Construct pUC-H1PF1 was generated to contain the sequence coding for the FLAG epitope and the kanamycin resistance cassette, and was used as the template to amplify the DNA fragments for homologous targeting in *Salmonella *ST14028s strain [43].

The primers used to construct the tagged mutants are listed in Table 3. For each tagged mutant, a pair of primers was designed to amplify the FLAG epitope and kanamycin resistance gene coding sequences using pUC-H1PF1 as the template [43]. The FLAG epitope is an octapeptide tag (N-DYKDDDDK-C) that has been widely used for tagging a protein, which in turn can be detected and studied using the anti-FLAG antibody [21]. The 3' ends of the forward and reverse primers were complementary to the first 20 nt of the FLAG tag sequence and the pUC-H1PF1 sequence immediately downstream from the sequence of the kanamycin resistance cassette, respectively. The 5' ends of the forward and reverse primers were designed to be complementary to the last 80 nt of each tagged ORF, not including the stop codon, and to the 80 nt immediately downstream of the stop codon, respectively. The resulting PCR products were transformed into *Salmonella *ST14028s strain carrying plasmid pKD46. The tagged mutants were constructed using the λRed recombinase method [44], following the procedures as described previously [45]. The tagged mutants were selected in the presence of kanamycin and further confirmed by PCR.

**Table 3:** The primers used to construct the tagged strains T-prgI, T-sipA, T-sipB, T-sopE2, T-spaO, and T-sptP.

ORFs	Upstream primer	Downstream primer
*prgI*	5'ATAACTTGTACCGTAACGCGCAA TCGAACACGGTAAAAGTCTTTAAG GATATTGATGCTGCCATTATTCAGA ACTTCCGTGATTACAAGGATGACG ACGA 3'	5'GACCTGATATTGACCGCCTGCCC TATAACGGCATTCTCAGGGACAAT AGTTGCAATCGACATAATCCACCTT ATAACTGACATATGAATATCCTCCT TAGTT 3'

*sipA*	5'GCCCGGCTTACGAGTCATACCTG AATAAACCTGGCGTGGATCGGGTT ATTACTACCGTTGATGGCTTGCACA TGCAGCGTGATTACAAGGATGACG ACGA 3'	5'ACATCAACGGCAATACAAGAGG TGATCACTTTTTTGACTCTTGCTTCA ATATCCATATTCATCGCATCTTTCC CGGTTAACATATGAATATCCTCCTT AGTT 3'

*sipB*	5'CGGAACTGCAAAAAGCCATGTCT TCTGCGGTACAGCAAAATGCGGAT GCTTCGCGTTTTATTCTGCGCCAGA GTCGCGCAGATTACAAGGATGACG ACGA 3'	5'GAATGATTATTTAAATAAGCGGC GGGATTTATTCCCACATTACTAATT AACATATTTTTCTCCCTTTATTTTGG CAGTTTCATATGAATATCCTCCTTA GTT 3'

*sopE2*	5'ATAAGGGGACGATGCCAACGCC ACAACAATTTCAGTTAACTATAGA AAATATTGCGAATAAGTATCTTCAG AATGCCTCCGATTACAAGGATGAC GACGA 3'	5'TCGCCATAAAAACGAATATATAG TTTCAGAAAATCTGCTATTAATTCA TATGGTTAATAGCAGTATTGTATTT ACTACCACATATGAATATCCTCCTT AGTT 3'

*spaO*	5'ATGAATGACACCTTAGGCGTTGA GATCCATGAATGGCTGAGCGAGTC TGGTAATGGGGAAGATTACAAGGA TGACGACGA 3'	5'CCTGACGCAATAATAAATGGCAA CAGGGTGGAAAATGCCAGTAAGGC AATTAATGAGATACATATGAATAT CCTCCTTAGTT 3'

*sptP*	5'GCCTCACAGTTTGTACAACTAAA AGCAATGCAAGCCCAGTTGCTTAT GACGACGGCAAGCGATTACAAGGA TGACGACGA 3'	5'CAAATAATTATACAGAAATAGCT TACTTTCAGATAGTTCTAAAAGTAAG CTATGTTTTTACATATGAATATCCTC CTTAGTT 3'

Once homologous recombination was confirmed, the tagged mutations were transduced into fresh cultures of ST14028s by transduction using phage P22, and P22-free colonies were selected, following procedures described previously [46,47]. To generate non-polar strains, each of the tagged mutants was transformed with plasmid pCP20 by electroporation. The non-polar strains were selected for their sensitivity to kanamycin and further confirmed using PCR. The regions for the tagged ORFs in all the generated tagged strains (i.e. the homologous recombinant mutants, P22-trasduced mutants, and the non-polar mutants) were sequenced to confirm that no other mutations were found in these regions.

### *In vitro *studies of the expression of the tagged SPI-1 proteins

Colonies of tagged strains were inoculated in 1 ml of LB broth and cultured at 250 RPM and 37°C for 16 hours. The bacterial cultures were used to prepare for the pellet and supernatant samples for Western analysis.

To study the effect of different culture conditions on the protein expression *in vitro*, 20 μl of overnight bacterial cultures were inoculated into 1 ml of antibiotic-free LB and shaken at 250 RPM and 37°C for 4 hours to reach the late log phase. The bacterial cultures were centrifuged at 5,000 × g for 5 minutes. To study the effect of pH, the pelleted bacteria were re-suspended in 1 ml of fresh LB broth (control, pH7.0) or 1 ml of LB broth with pH 3.0, 5.0, 7.2, and 8.4, respectively, and shaken at 250 RPM and 37°C for additional 6 hours, and then collected. To study the effect of osmolarity, the pelleted bacteria were re-suspended in 1 ml of NaCl-free LB broth supplemented with 0, 42.5, 85, 170, 340, and 680 mM sodium chloride, respectively, and then shaken at 250 RPM and 37°C for additional 6 hour, and were collected. Regular LB broth, which contained 170 mM NaCl, was used as the control. To study the effect of butyrate, the pelleted bacteria were re-suspended in 1 ml of fresh LB broth (control) or 1 ml of LB broth containing 10 mM sodium butyrate and shaken at 250 RPM and 37°C for additional 6 hours, and then collected. To study the effect of oxygen ventilation, the pelleted bacteria were re-suspended in 1.5 ml of fresh LB broth. One group of bacteria was shaken at 250 RPM and 37°C for additional 6 hours with good aeration (control) while another group of bacteria was transferred into 1.5 ml microcentrifuge tubes with their covers closed tightly, and incubated at 37°C without shaking for additional 6 hours.

### Preparation of culture supernatants and cell extracts from bacterial grown *in vitro *under different conditions

To prepare protein samples from the pellets of bacterial cultures, the cultures (1 ml) were centrifuged at 5,000 × g and 4°C for 10 minutes. The pellets were re-suspended in 200 μl of bacterial lysis buffer (8 M urea, 2% chaps, and 10 mM Tris, pH8.0). The bacterial suspension was sonicated for 15 seconds three times with an interval of 30 seconds, centrifuged at 5,000 × g and 4°C for 10 minutes, and then transferred into new tubes for Western analysis. To prepare secreted protein samples, 0.5 ml of ice-pre-cooled 25% TCA was added into the supernatants of the bacterial cultures (1 ml). The mixture was incubated at 4°C for 15 minutes, and then centrifuged at 15,000 × g and 4°C for 10 minutes to precipitate soluble proteins. The pellets were washed with acetone twice, dried in air for 30 minutes, and then re-suspended in phosphate buffered saline (PBS) for Western analysis [45,48]. The protein concentrations of the pellet and soluble proteins were determined by Bradford Method on a micro-plate reader with absorbance at 495 nm using a standard curve of BSA concentrations.

### *In vivo *studies

Female BALB/c and SCID mice (6–8 weeks old) were obtained from Jackson Laboratory (Bar Harbor, ME). Mice were kept in sterilized, filter-topped cages, handled in laminar hoods, and fed autoclaved food and water under specific pathogen-free (SPF) conditions at our animal facilities. Sentinel animals were routinely tested for common pathogens. The protocols used were in compliance with the guidelines and policies of the Animal Care and Use Committee (ACUC) of the University of California at Berkeley. Overnight bacterial cultures were serially diluted to suitable CFU/ml in PBS for infection. To assess the virulence of the tested strains, groups of five mice were either inoculated intragastrically with 5 × 10^6 ^CFU per BALB/c mouse and 1 × 10^3 ^CFU per SCID mouse or intraperitoneally with 1 × 10^2 ^CFU per BALB/c mouse and 1 × 10^1 ^CFU per SCID mouse. Mice were monitored during the course of infection, and those animals that exhibited extreme stress or became moribund were euthanized [45,48]. For organ colonization and *in vivo *experiments, groups of five mice were inoculated intraperitoneally with 1 × 10^5 ^or 1 × 10^7 ^CFU per BALB/c mouse or 1 × 10^2 ^or 1 × 10^4 ^CFU per SCID mouse of the bacterial strains, and were euthanized at 5 days or 18 hours after inoculation, respectively. Mice (5 animals per group) were also inoculated intragastrically with 1 × 10^5 ^or 1 × 10^8 ^CFU per BALB/c mouse or 1 × 10^2 ^or 1 × 10^4 ^CFU per SCID mouse of the bacterial strains and were euthanized at 7 days or 24 hours after inoculation, respectively. Organs were collected and homogenized in cold PBS. An aliquot of homogenate was used to determine its CFU/ml by serial dilution with PBS and plating on LB agar plates [45,48].

To prepare protein extracts for Western analyses, the homogenates of the spleen samples were centrifuged at 9,000 × g and 4°C for 10 minutes. The pellets from the spleen were resuspended in 0.5 ml of cold lysis buffer (120 mM NaCl, 4 mM MgCl2, 20 mM Tris/HCl, pH 7.5, 1% Triton-X100) supplemented with protease inhibitors (complete EDTA-free cocktail, Roche), incubated at 4°C for 1 hour, centrifuged at 18,000 × g and 4°C for 10 minutes. The pellets that contained the bacteria were resuspended in PBS for Western analyses [45,48]. For the cecum samples, the homogenates were incubated on ice for 10 minutes. The upper clear suspensions were transferred and centrifuged at 15,000 × g and 4°C for 10 minutes. The pellets were washed in PBS, centrifuged at 18,000 × g and 4°C for 10 minutes, and resuspended in PBS for Western analyses [45,48].

### Western analyses

The denatured polypeptides from bacterial lysates were separated on SDS-containing 10–12% polyacrylamide gels cross-linked with *N*,*N*"-methylenebisacrylamide, transferred electrically to nitrocellulose membranes, and reacted in an enzyme-linked immunoassay with anti-mouse IgG conjugated with alkaline phosphatase in addition to the antibodies against the FLAG sequence (Sigma, St Louis, MO) and *Salmonella *DnaK protein [45,49]. The membranes were subsequently stained with a chemiluminescent substrate with the aid of a Western chemiluminescent substrate kit (Amersham Inc, GE Healthcare) and quantitated with a STORM840 phosphorimager. Quantitation was performed in the linear range of protein detection. Normalization of samples was also carried out by loading total protein extracted from the same CFU (e.g. 5 × 10^7 ^CFU) of bacteria in each lane.

### Determination of the CFU counts

An aliquot of tissue homogenate or bacterial culture was used to determine its CFU/ml by serial dilution with PBS and plating on LB agar plates [45,48]. The bacteria were enumberated after overnight incubation. Each sample was analyzed in triplicate and the analysis was repeated at least twice. The CFU of the sample was expressed as the average of the values obtained. The concentrations of bacteria were recorded as CFU/ml of organ homogenate or culture. The limit of bacteria detection in the organ homogenates was 10 CFU/ml. Those samples that were negative at a 10^-1 ^dilution were designated a value of 10 (10^1^) CFU/ml.

## Authors' contributions

HG, JS, YB, LM, KK, YY, FL, and SL conceived the study, performed the research, analyzed the results, and wrote the paper. All authors read and approved the final manuscript.
